# A rare gene variation cap +1 (A>C) (HBB: c. −50A>C) associated with codon 5 (‐CT) (HBB: c.17_18delCT) mutation in Syrian family

**DOI:** 10.1002/mgg3.1602

**Published:** 2021-01-24

**Authors:** Hossam Murad, Faten Moassas, Nour A. L. Fakseh

**Affiliations:** ^1^ Molecular Biology and Biotechnology Department Human Genetics Div Atomic Energy Commission of Syria Damascus Syria

**Keywords:** Rare silent mutation, Syria; CAP+1 [A>C], β‐Thalassemia (β‐thal)

## Abstract

**Background:**

CAP+1 [A>C] (HBB:c.‐50A>C) is a rare silent β‐thalassemia (β‐thal) mutation. Carrier individuals of this mutation show borderline hemoglobin (Hb), mean corpuscular volume (MCV) and Hb A2 levels. This mutation was previously reported in combination with different β‐thalassemia mutations, leading to variable phenotypes.

**Case presentation:**

Here, we describe for the first time the combination of silent CAP+1 [A>C] (HBB:c.‐50A>C) mutation with β^0^ codon 5 [‐CT] (HBB:c.17_18delCT) mutation in a Syrian proband, leading to beta thalassemia intermedia (TI).

**Conclusions:**

The compound heterozygotes of the silent CAP+1 (A>C) together with another severe beta gene mutation, are phenotypically severe enough to present at an early age and require appropriate therapeutic modalities.

## Graphical abstract

CAP+1 [A>C] (HBB:c.‐50A>C) is a rare silent β‐thalassemia (β‐thal) mutation. We have for the first time the combination of CAP+1 [A>C] (HBB:c.‐50A>C) mutation with β0 codon 5 [‐CT] (HBB:c.17_18delCT) mutation in a Syrian proband. This combination leads to beta thalassemia intermedia (TI).

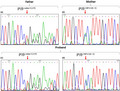

## 1 INTRODUCTION

Beta thalassemia (β‐thal) is the most common blood disease in Syria (Jarjour et al., 2014; Murad et al., 2014). It described by reduction or absence of beta globin chain synthesis (Galanello & Origa, 2010). Most beta thalassemia mutations are caused by point mutations, insertions or small deletions within the coding regions, the exon‐intron junctions and substitution within the TATA box of the β‐globin gene promoter (Cao et al., 2011; Ropero et al., 2017). There are certain “silent” β‐thalassemia mutations in which red‐cell indices are normal and Hb A2 is not raised in carriers (Aslan, 2016; Vinciguerra et al., 2018). CAP+1 (A>C) transversion is one of these mutations which was previously found in Asian Indians (Wong et al., 1987). This mutation was described in a homozygous individual who presented a β‐thalassemia trait with a Hb A2 level of 4.1%. While, presence of this variant with different β‐thalassemia mutations as a compound heterozygous state, showed a heterogeneous clinical presentation from nontransfusion‐dependent to transfusion‐dependent thalassemia patients (Garewal et al., 2007).

Herein, we describe for the first time the combination of CAP+1 [A>C] (HBB:c.‐50A>C) mutation with β^0^ codon 5 [‐CT] (HBB:c.17_18delCT) mutation in a Syrian proband which leads to β‐thalassemia intermedia (TI).

## 2 METHODS

### 2.1 Ethical statement

This study has been approved by the Institutional Review Board of the Atomic Energy Commission of Syria (AECS).

### 2.2 Patient

A 12‐year‐old female Syrian proband, was referred to our center AECS‐ in Damascus for confirmation of her status for β‐thalassemia. The parents were nonconsanguineous. Her history revealed pallor and anemia. The electropherogram revealed a low level of (Hb A1) 52.6%, high level of (Hb F) 42.5% and (Hb A2) was 4.9%. The proband received erythrocyte transfusions at the age of 2 years. She underwent splenectomy at 8 years. She requires blood transfusion every 40 days. Her father had a classic clinical picture of β‐thalassemia trait. Her mother had normal indices with a borderline of (Hb A2) 3.5%. Both parents had no splenomegaly and hepatomegaly and had never been transfused. All hematological data have been summarized in Table 1.

**TABLE 1:** The hematological and molecular data of the family.

Parameters	Father	Mother	Proband
Sex‐age (years)	M−38	F−35	F−12
Hb (g/dL)	11.5	14.1	9.3
RBC (10^12^/L)	5.8	5.68	2.71
MCV (fL)	60.1	77.1	91.5
MCHC (g/dl)	32.4	32.2	34.1
MCH (pg)	19.5	24.8	31.2
Hb A1 (%)	92.2	96.1	52.6
Hb A2 (%)	5.8	3.5	4.9
Hb F (%)	2	0.4	42.5
α Genotype	αα/αα	αα/–α^3.7^	αα/–α^3.7^
β Genotype	β^A^/β^codon 5[‐CT]^	β^A^/β^CAP +1 [A>C]^	β^CAP +1 [A>C]^/β^codon 5[‐CT]^

Abbreviations: Hb, hemoglobin; MCH, mean corpuscular Hb; MCHC, mean corpuscular Hb concentration; MCV, mean corpuscular volume; RBC, red blood cell count.

### 2.3 Analysis of hematological parameters and hemoglobin components

An automated differential cell counter (ABX Micros ES60; HORIBA ABX SAS, Montpellier, France) was used to obtain the hematological parameters of the parents and proband. A Capillarys 2 system (Sebia, Lisses, France) was used to analyse Hemoglobin electrophoresis (Hb).

### 2.4 DNA sample preparation & Sequencing

QIAamp DNA Blood Mini kit (Qiagen, Germany) was used to isolate the genomic DNA from peripheral the blood of both parents and proband according to the manufacturer's instructions. Purified gDNA was run on a 0.8% agarose gel.

Polymerase chain reaction (PCR) method was used to determine the genotype of HBB gene. The appropriate primers used for exon 1, exon 2 and exon 3 of β‐globin gene included the promoter, first intron, 5` and 3` untranslated region (UTR) sequences as previously reported (Yassin et al., 2013). The entire human HBB gene was sequenced using an ABI PRISM 310‐DNA Analyzer (Applied Biosystem, Foster City, CA, USA) as reported previously (Murad et al., 2014, 2018).

### 2.5 Reverse hybridization assay

Reverse hybridization assay (ViennaLab Diagnostics Gmb Vienna, Austria) used to to detect α‐globin gene deletions. To investigate of the increased level of Hb F in the proband, PCR‐RFLP (restriction fragment length polymorphism) was used for the detection of ‐158Gγ (C→T) Xmn1 polymorphism using the Xmn1 restriction enzyme with specific primers (Rujito et al., 2016).

## 3 RESULTS

### 3.1 DNA sequencing & Reverse hybridization assay

**FIGURE 1:**
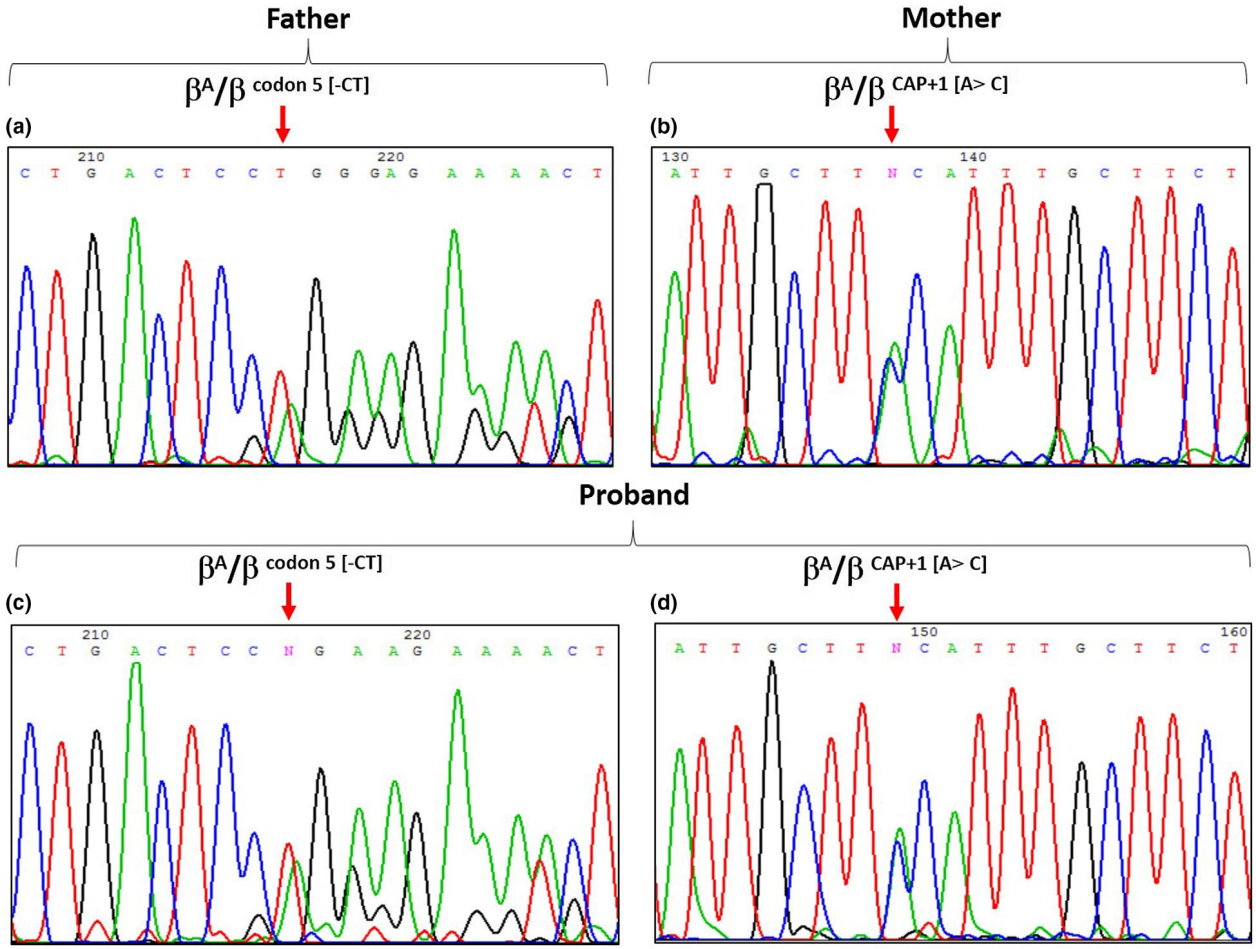
Direct sequencing analysis revealed the PCR fragment on the β‐globin gene. (a), (c) the arrows indicate the [‐CT] deletion at codon 5 in the β‐globin gene for the father and the proband respectively; (b), (d) the arrows indicate the CAP+1 substitution at the −50 in the β‐globin gene for the mother and the proband respectively.

Direct sequencing of the β‐globin gene confirmed that the mother was found to be heterozygous for the CAP+1 [A>C] (HBB:c.‐50A>C) variation, and the father was heterozygous for the β^0^ codon 5 [‐CT] (HBB:c.17_18delCT) mutation. The chromatogram of the proband sample revealed that the proband had CAP+1 [A>C] (HBB:c.‐50A>C) and β^0^ codon 5 [‐CT] (HBB:c.17_18delCT) mutation in a heterozygous state (Figure 1). Reverse hybridization assay to characterize the α‐globin gene deletion revealed that, the proband and his mother had the rightward (−α3.7) deletion in a heterozygous state. XmnI polymorphism at −158 to the Gγ‐globin gene was absent (XmnI [−/−]) for all samples.

## 4 DISCUSSION

CAP+1 (A>C) is known as a silent HBB variation that was first reported in an Asian Indian patient (Wong et al., 1987). The carriers of this variation CAP+1 (A>C) have been shown to have borderline hematologic profiles (Garewal et al., 2007; Rangan et al., 2011). In our case, the mother was found to be carrier of the CAP+1 [A>C] variation, and her hematologic parameters were (MCV) 77.1 fl, (MCH) 24 pg, and Hb A2 level was 3.5%.

Compound heterozygosity of the silent CAP+1 (A>C) variation with different β‐thalassemia mutations showed a heterogeneous clinical presentation (Garewal et al., 2007). The combination of the silent CAP+1 (A>C) variation with different mutations has been previously reported. The combination of CAP+1 (A>C) variation with β^0^ IVS‐I‐5 (G>C) mutation was relatively leading at 85% of cases to the beta thalassemia major (TM). In the other hand, 50% of cases with CAP+1 (A>C) / codons 41/42 (‐TTCT) mutation had beta thalassemia intermedia (TI), while, the remains combinations with other mutations had beta thalassemia major (TM). Our proband had silent CAP+1 [A>C] variation and β^0^ codon 5 [‐CT] mutation in a heterozygous state. We propose that, this combination leads to beta thalassemia intermedia (TI), wherein, the proband requires blood transfusion every 40 days.

Complete inhibition of β‐chain synthesis by the β^0^ Codon 5 [‐CT] mutation was known by the creation of a premature termination signal at codon 21 (Kollia et al., 1989). In our case, the combination of a silent HBB mutation CAP+1 (A>C) with β^0^ codon 5 [‐CT] mutation was reported for the first time, and it led to transfusion‐dependent thalassemia in the patient.

## 5 CONCLUSIONS

In this case, we report for the first time the silent CAP+1 (A>C) (HBB: c.‐50A<C) variation in a Syrian family. The compound heterozygotes of the silent CAP+1 (A>C) variation and another severe beta gene mutation, are phenotypically severe enough to present at an early age and require appropriate therapeutic modalities.

## CONFLICT OF INTEREST

The authors report no conflicts of interest. The authors alone are responsible for the content and writing of this article.

## Data Availability

Not applicable. All data generated or analyzed during this study are included in this published article.
